# Acute stress and hippocampal output: exploring dorsal CA1 and subicular synaptic plasticity simultaneously in anesthetized rats

**DOI:** 10.1002/phy2.35

**Published:** 2013-07-21

**Authors:** Matthew J MacDougall, John G Howland

**Affiliations:** Department of Physiology, University of SaskatchewanGB33, Health Sciences Building, 107 Wiggins Road, Saskatoon, Saskatchewan, Canada, S7N 5E5

**Keywords:** Hippocampus, in vivo electrophysiology, late-developing potentiation, long-term potentiation, paired-pulse facilitation

## Abstract

The Cornu Ammonis-1 (CA1) subfield and subiculum (SUB) serve as major output structures of the hippocampal formation. Exploring forms of synaptic plasticity simultaneously within these two output regions may improve understanding of the dynamics of hippocampal circuitry and information transfer between hippocampal and cortical brain regions. Using a novel dual-channel electrophysiological preparation in urethane-anesthetized adult male Sprague-Dawley rats in vivo, we examined the effects of acute restraint stress (30 min) on short- and long-term forms of synaptic plasticity in both CA1 and SUB by stimulating the CA3 region. Paired-pulse facilitation was disrupted in SUB but not CA1 in the dual-channel experiments following exposure to acute stress. Disruptions in CA1 PPF were evident in subsequent single-channel experiments with a more anterior recording site. Acute stress disrupted long-term potentiation induced by high-frequency stimulation (10 bursts of 20 pulses at 200 Hz) in both CA1 and SUB. Low-frequency stimulation (900 pulses at 1 Hz) did not alter CA1 plasticity while a late-developing potentiation was evident in SUB that was disrupted following exposure to acute stress. These findings highlight differences in the sensitivity to acute stress for distinct forms of synaptic plasticity within synapses in hippocampal output regions. The findings are discussed in relation to normal and aberrant forms of hippocampal-cortical information processing.

## Introduction

The mammalian hippocampal formation consists of several anatomically distinct subregions including the entorhinal cortex, dentate gyrus, hippocampus proper (Cornu Ammonis [CA] subfields, CA3 and CA1), and subiculum (SUB) (O'Mara et al. 2001; Andersen et al. 2006; van Strien et al. 2009). Standard anatomical views hold that a number of major glutamatergic pathways direct information flow through the hippocampal formation (Andersen et al. 2006; van Strien et al. 2009). Accordingly, highly integrated sensory information from entorhinal cortex (layer II) arrives at dentate gyrus via the perforant path or the CA3 and CA1 regions via the temporoammonic pathway (Behr et al. 2009; van Strien et al. 2009). Dentate gyrus granular cells direct this information to CA3 neurons via the mossy fibers which in turn project to the CA1 region through the Schaffer collaterals. Lastly, CA1 pyramidal cells project either directly back to the entorhinal cortex or through a topographically organized projection to SUB (O'Mara et al. 2001; Andersen et al. 2006). The majority of subicular cells conserve their topographic input along the transverse axis from CA1 and transmit information to the deep layers (layers V and VI) of entorhinal cortex (van Strien et al. 2009). Thus, both CA1 and SUB function as major output structures for the hippocampal formation and are therefore integral for hippocampal-cortical information processing. Although the traditional polysynaptic description of hippocampal formation circuitry is considered unidirectional, reciprocal back-projections for all of the major pathways have been demonstrated (Amaral and Witter 1989; Buckmaster et al. 1993; Scharfman 1994; Naber et al. 2001a; Commins et al. 2002; Witter 2007), as have other intra- and para-hippocampal projections (Amaral and Witter 1989; Naber et al. 2001b; Andersen et al. 2006; van Strien et al. 2009).

Patterns of synaptic plasticity such as paired-pulse facilitation (PPF), long-term potentiation (LTP), long-term depression (LTD), and late-developing potentiation are observed at synapses within the hippocampal formation and the mechanisms governing these forms of plasticity have been implicated in cognitive processes such as spatial learning and memory (O'Keefe and Nadel 1978; Martin et al. 2000; Malenka and Bear 2004; Massey and Bashir 2007; Behr et al. 2009; Collingridge et al. 2010; Klug et al. 2012). Alterations in synaptic plasticity in both the CA1 and SUB are observed following acute stress, effects that are proposed to underlie the effects of acute stress on spatial learning and memory (Kim and Diamond 2002; Joels et al. 2006; Diamond et al. 2007; Howland and Wang 2008; Cazakoff et al. 2010; Collingridge et al. 2010; Segal et al. 2010). For example, experiments conducted with field potential recordings have revealed that acute stress disrupts PPF and LTP in the CA1 and SUB (Shors and Thompson 1992; Diamond and Rose 1994; Kim et al. 1996; Cazakoff and Howland 2010; MacDougall and Howland 2013) via glucocorticoid receptor activation (Xu et al. 1998; Cazakoff and Howland 2010; MacDougall and Howland 2013). In addition, acute stress enables LTD in the CA1 region (Xu et al. 1997; Wong et al. 2007) and disrupts late-developing potentiation in SUB (MacDougall and Howland 2013), effects also mediated by glucocorticoid receptor activation (Xu et al. 1998; MacDougall and Howland 2013). To our knowledge, all previous studies examining the effects of acute stress on hippocampal synaptic plasticity in vivo have recorded from one site; therefore, the effect of acute stress on synaptic plasticity in multiple subregions of the hippocampal formation circuitry remains unknown (Joels et al. 2012). A recent study using simultaneous single unit recordings from the CA1, CA3, and dentate gyrus regions in the same rat provided evidence of differential modulation of these regions by acute stress, although synaptic plasticity was not measured in this study (Passecker et al. 2011). Thus, it is likely that acute stress may differentially modulate patterns of synaptic plasticity in discrete subregions of the hippocampal formation.

**Figure 1:**
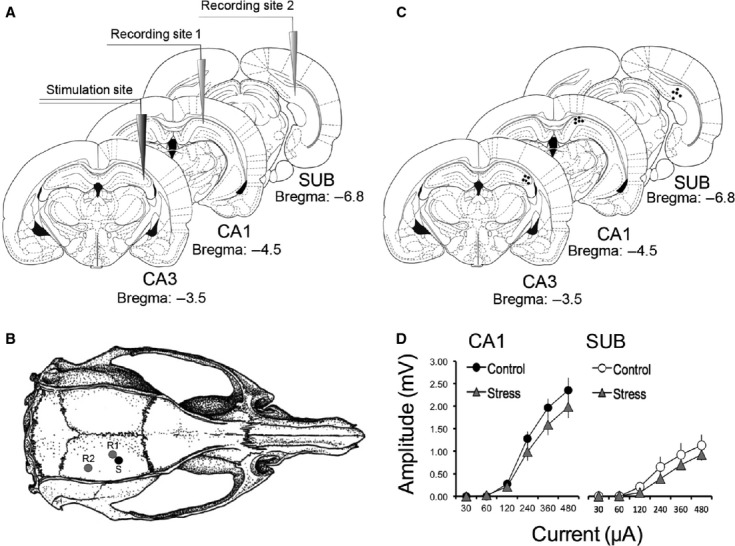
(A) A schematic of the experimental design with stimulating electrode placed in dorsal CA3 and recording electrodes placed in dorsal CA1 and subiculum (SUB). (B) A rendering of a rat skull and the positions of the bored holes for the placements of the stimulating (S) and the two recording (R1 and R2) electrodes. Adapted from Paxinos and Watson (1997). (C) Representative electrode placements in the three regions of interest as indicated by black dots. (D) Input/output curves for CA1 (left) and SUB (right) for control and stressed rats obtained prior to the initial PPF recordings.

Given the established sensitivity of the hippocampal formation to acute stress, the aim of the present study was to examine the effects of acute restraint stress on distinct forms of synaptic plasticity as they exist within dorsal CA1 and SUB concurrently. Using a novel dual-channel recording configuration that allowed for the simultaneous recording of evoked potentials in the CA1 and SUB simultaneously in vivo (Fig. 1), we induced field excitatory postsynaptic potentials (fEPSPs) in CA1 and SUB by stimulating the CA3 Schaffer collaterals. For the first time, we demonstrate simultaneous fEPSPs in the CA1 and SUB following CA3 stimulation and differential effects of acute stress on concurrent patterns of short- and long-term synaptic plasticity in the CA1 and SUB.

## Material and Methods

### Subject

Adult male Sprague-Dawley rats (>300 g; Charles River Laboratories, Quebec, Canada) were pair housed in plastic cages with ad libitum access to food and water. Rats were housed under a 12:12 h light/dark cycle (lights on at 07:00) in a temperature and humidity controlled vivarium. Experimentation was conducted during the light phase. After arrival at the facility, rats were given at least 5 days to acclimatize before experiments were initiated. All experiments were conducted in accordance with the Canadian Council on Animal Care and were approved by the University of Saskatchewan Animal Research Ethics Board.

### In vivo electrophysiology

Rats were anesthetized using urethane (1.5–2.0 g/kg, i.p.) and placed in a stereotaxic frame (David Kopf, CA). A grounded homeothermic temperature control unit (Harvard Instruments, MA) was used to maintain the rectal temperature of the rats at 37°C ± 1°C during the experimental sessions. For all dual-channel experiments, monopolar recording electrodes (insulated platinum iridium wire, 125 μm; AM Systems, WA) were lowered into the dorsal CA1 (AP = −4.5 mm, ML = 2.5 mm, DV = −2.5 mm) and dorsal SUB (AP = −6.8 mm, ML = 4.00 mm, DV = −2.5 mm; Fig. 1A and B). A stimulating electrode (NE-100X; Rhodes Medical Instruments, CA; tip separation = 0.5 mm) was lowered into the dorsal CA3 region (AP = −3.5 mm, ML = 3.5 mm, DV = −2.5 mm; Fig. 1A–C). For all single-channel experiments, the methods were identical to those of the dual-channel experiments with the exception of electrode placements; for all single-channel experiments, one recording electrode was lowed into dorsal CA1 region (AP = −3.0 mm, ML = 3.0 mm, DV = −2.5) and the stimulating electrode was lowered into the dorsal CA3 subfield (AP = −3.5 mm, ML = 3.5 mm, DV = −2.5 mm; Fig. 4A–C). A reference wire for the recording electrode(s) was secured to the skull anterior to bregma with a jeweller's screw. Both CA1 and SUB fEPSPs were evoked by stimulation of CA3 Schaffer collaterals (pulse width = 0.12 msec, 200 μA, 0.2 Hz) and were recorded at varying depths. Final electrode placements were determined by maximal field responses and the electrical current was adjusted in all experiments to elicit fEPSPs of 50–60% of the maximal responses (Figs. 1D and 4D). Recordings were initiated 15–20 min following optimization of electrode placements.

At the start of each recording session, the amplitudes of fEPSPs were established in each region at various current intensities (30, 60, 120, 240, 360, and 480 μA) and input/output curves were calculated. Paired-pulse facilitation (PPF) was then measured by delivering five pairs of pulses to CA3 at interpulse intervals of 25, 50, 100, and 200 msec. Immediately following PPF, baseline fEPSPs were obtained by administering stimulation (0.067 Hz) until a stable baseline was achieved for 20 min. Two tetanus protocols were used for the dual-channel recordings: the HFS protocol consisted of 10 bursts of 20 pulses at 200 Hz with an interburst interval of 2 sec (Commins et al. 1998b; MacDougall and Howland 2013) while the LFS protocol consisted of 900 pulses delivered at 1 Hz (Anderson et al. 2000; MacDougall and Howland 2013). A HFS of 100 Hz for 1 sec was used in single-channel recordings to precisely replicate previous experiments (Cazakoff and Howland 2010). In all experiments (i.e., single and dual channel), the baseline stimulation frequency was resumed following the tetanus and responses were recorded for 60 min after which the input/output curves and PPF were reexamined as described above. Due to technical problems with the software for recordings, the PPF values for two rats could not be included in the final analysis.

### Acute stress protocol

Acute stress was accomplished by immobilizing rats in a Plexiglas restraint tube (544-RR, Fisher Scientific, Ottawa, ON, Canada) in a brightly lit novel room for 30 min. We have previously demonstrated that this behavioral stress protocol significantly elevates circulating levels of corticosterone in rats (MacDougall and Howland 2013). Rats exposed to this form of acute stress also consistently displayed high levels of urination, defecation, and piloerection. All rats were anesthetized immediately following acute stress and mounted on a stereotaxic frame in preparation for electrophysiological recordings.

### Histology

Following the recordings, electrolytic lesions were created by administering direct current (0.2 mA, 20 sec) through the electrodes. Rats were then transcardially perfused with 30 mL of physiological saline and their brains removed and stored in a 10% formalin–10% sucrose solution. Brains were sectioned using a sliding microtome and electrode placements were verified (Figs. 1C and 4B) with the aid of a rat brain atlas (Paxinos and Watson 1997) and compound light microscope (Fisher Scientific).

### Statistical analysis

Statistical tests were conducted using SPSS Version 18 (IBM, Armonk, NY) for Windows and Graphpad Prism 5.0. All descriptive values are reported as means ± standard error of the mean (SEM). *P* values of less than or equal to 0.05 were considered statistically significant. PPF is expressed as percent change in the second evoked fEPSP slope relative to the first fEPSP slope. We averaged the short (25 and 50 msec) and long (100 and 200 msec) latency interpulse intervals, as significant differences did not exist between these intervals (*P* > 0.05). Omnibus repeated measures analyses of variance (ANOVA) revealed no significant effect of Tetanus for PPF values; therefore, PPF data were combined for HFS and LFS groups in all analyses. The magnitude of long-term plasticity was normalized and expressed as the percent change in fEPSP slope from the 20 min baseline. For each group, comparisons between the average fEPSP slope for the last 5 min of baseline and the last 5 min of the 1 h decay period were made using paired sample *t*-tests. Between group comparisons were made using *t*-tests or ANOVA as appropriate.

## Results

### Effects of acute stress on PPF in CA1 and SUB elicited by CA3 stimulation

**Figure 2:**
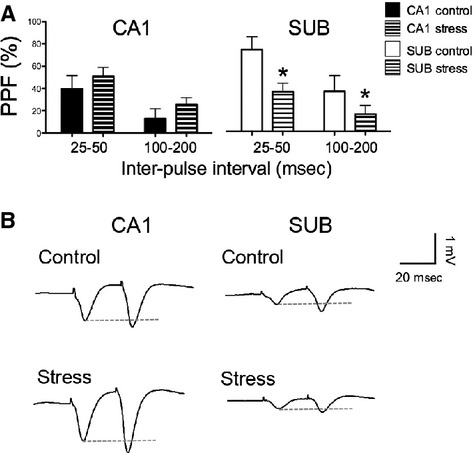
Paired-pulse facilitation (PPF). (A) Pretetanus PPF values for the averaged 25–50 msec and 100–200 msec interpulse intervals in CA1 and subiculum (SUB) for control (*n* = 9; solid bars) and stressed (*n* = 12; striped bars) rats. (B) Representative field excitatory postsynaptic potential (fEPSP) traces as measured simultaneously from CA1 and SUB following the stimulation of CA3 for control and stress conditions. *Significant difference between control and stress groups for SUB.

Stimulation of the CA3 region evoked robust field potentials in the CA1 and SUB subregions of the hippocampus (Figs. 1, 2). The maximal amplitude of the field potential was greater in the CA1 (∼2 mV) than the SUB subregion (∼1 mV; Fig. 1D). The latency of the peak amplitude of the fEPSPs during the 5 min before the tetanus was delivered did not differ significantly between recording sites (CA1 = 8.20 ± 0.30 msec; SUB 8.02 ± 0.35 msec). Before the tetanus was delivered, PPF was observed in both CA1 (25–50 msec: 39.60 ± 16.93%; 100–200 msec: 13.06 ± 11.60%) and SUB (25–50 msec: 74.28 ± 16.11%; 100–200 msec: 36.37 ± 20.05%) in control animals (*n* = 9) in response to CA3 stimulation (Fig. 2A and B). Rats exposed to acute stress (*n* = 12) had levels of PPF similar to controls in the CA1 (25–50 msec: 51.05 ± 10.91%; 100–200 msec: 25.74 ± 6.40%) and while PPF was reduced by approximately half in SUB (25–50 msec: 35.92 ± 9.17%; 100–200 msec: 17.32 ± 10.27%; Fig. 2A and B). A repeated measures ANOVA on the pretetanus PPF values revealed a significant main effect of Interval (*F*_1,19_ = 31.46, *P* < 0.001) but no significant main effect of Stress (*F*_1,19_ = 0.42, *P* = 0.53) or Brain Area (*F*_1,19_ = 0.75, *P* = 0.40). A significant interaction between Brain Area and Stress was noted (*F*_1,19_ = 5.34, *P* = 0.03), whereas other interactions were not significant (statistics not shown). Post hoc analyses confirmed that acute stress reduced PPF in the SUB, but not CA1, subregion regardless of the PPF interval (*P* < 0.05).

Paired pulse facilitation was also measured after the 60 min posttetanus decay period for both CA1 and SUB. A repeated measures ANOVA on the pretetanus and posttetanus PPF values did not reveal a significant main effect of Time of PPF (i.e., pre- or posttetanus), although a significant main effect of Interval was observed (*F*_1,19_ = 51.09, *P* < 0.001). No other significant main effects or interactions for Brain Area or Stress were significant except for a significant Time of PPF by Interval interaction (*F*_1,19_ = 5.78, *P* = 0.03).

### High-frequency stimulation of CA3 induces LTP in CA1 and SUB that is disrupted by exposure to acute stress

**Figure 3:**
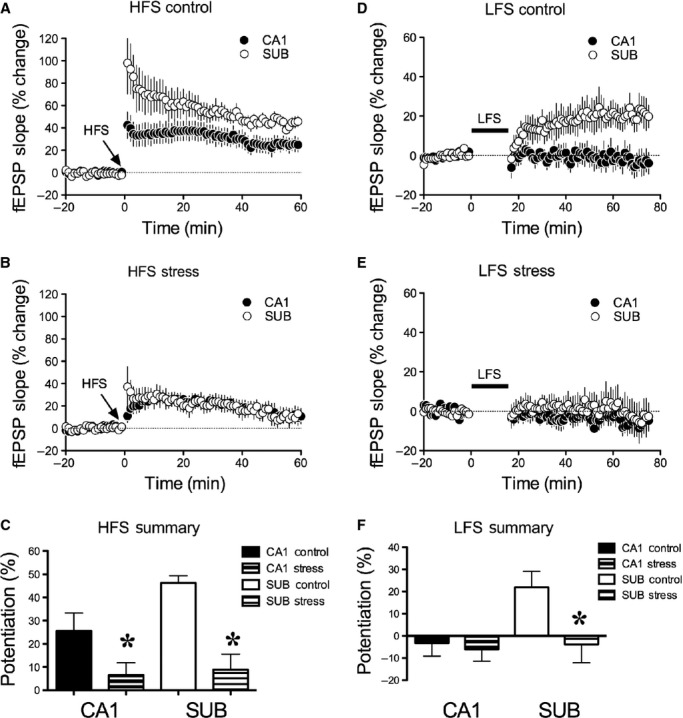
Long-term plasticity. (A) High-frequency stimulation (HFS)-induced long-term potentiation (LTP) is present in CA1 (black circle) and subiculum (SUB; open circles) under control conditions (*n* = 5). (B) Exposure to acute stress (*n* = 6) reduces the magnitude of HFS-induced LTP in CA1 (black circles) and SUB (open circles). (C) Summary of control and acute stress treatment on HFS-induced LTP in CA1 and SUB. (D) Low-frequency stimulation (LFS)-induced late-developing potentiation is present in SUB (open circles) but not CA1 (black circles) in control rats (*n* = 6). (E) Exposure to acute stress (*n* = 6) disrupts LFS-induced late-developing potentiation in SUB (open circles) but has no effect on CA1 (black circles) plasticity. (F) Summary of control and acute stress treatment following LFS in CA1 and SUB. *Significantly greater potentiation in the control than acutely stressed rats.

We observed that HFS of the CA3 subfield induced reliable LTP concurrently in both CA1 (25.51 ± 7.75%) and SUB (46.20 ± 3.16%; *n* = 5; Fig. 3A and C). Exposure to 30 min of restraint stress (*n* = 6) reduced the magnitude of this synaptic potentiation in both CA1 (6.53 ± 5.29%) and SUB (8.65 ± 6.72%) to levels that were not significantly different from baseline measurements (Fig. 3B and C; paired *t*-tests not shown). A repeated measures ANOVA revealed a significant main effect of Stress (*F*_1,9_ = 15.69, *P* = 0.003), and a main effect of Brain Area (*F*_1,9_ = 5.84, *P* = 0.039) and an interaction between Brain Area and Stress that was of borderline significance (*F*_1,11_ = 3.86, *P* = 0.081) (Fig. 3C). Inspection of the acute stress data revealed that LTP in the SUB was higher than in the CA1 region following HFS, an effect driven by the high level of potentiation in the nonstress condition.

### Low-frequency stimulation of CA3 has no effect on CA1 fEPSPs but induces a late-developing potentiation in SUB that is disrupted following exposure to acute stress

Low-frequency stimulation of the CA3 (*n* = 6) produced no consistent changes in fEPSP measurements within CA1 (−3.31 ± 4.80%) but induced late-developing potentiation in SUB (21.92 ± 5.86%; Fig. 3D and F). Exposure to 30 min of restraint stress (*n* = 6) disrupted the late-developing potentiation in SUB (−3.81 ± 8.38%; Fig. 3E and F) without effects on evoked fEPSPs in the CA1 region (0.24 ± 7.72%). Consistent with these observations, a repeated measures ANOVA revealed no significant main effect of Brain Area (*F*_1,10_ = 2.80, *P* = 0.13) or Stress (*F*_1,10_ = 2.30, *P* = 0.16) but a significant interaction between Brain Area and Stress (*F*_1,10_ = 5.35, *P* = 0.043) (Fig. 3F) following LFS. Post hoc analyses confirmed that the late-developing potentiation in SUB following LFS was significantly reduced in rats subjected to acute stress (Fig. 3F; *P* < 0.05).

### Exposure to acute stress disrupts PPF and LTP in more anterior zones of CA1 following stimulation of the CA3 Schaffer collaterals

**Figure 4:**
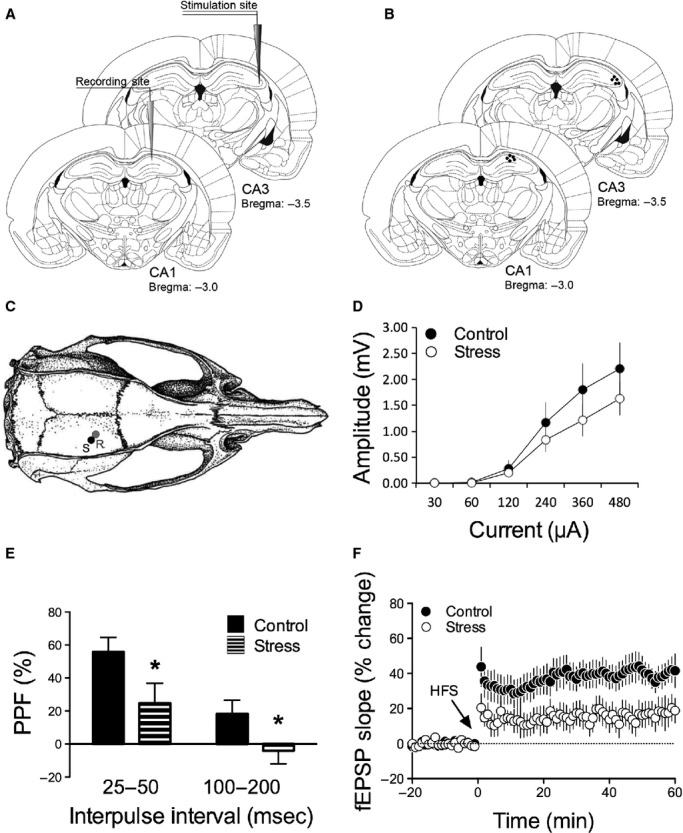
Single-channel recordings. (A) A schematic of the single-channel experimental design with stimulating electrode placed in dorsal CA3 and recording electrode placed in dorsal CA1. (B) Representative electrode placements in the two regions of interest as indicated by black dots. (C) A rendering of a rat skull and the positions of the bored holes for the placements of the stimulating (S) and the recording (R) electrodes. Adapted from Paxinos and Watson (1997). (D) Input/output curves for CA1 for control and stressed rats obtained prior to the initial PPF recordings. (E) Pretetanus PPF values for the averaged 25–50 msec and 100–200 msec interpulse intervals in CA1 for control (solid bars) and stressed (striped bars) rats. (F) High-frequency stimulation (HFS)-induced long-term potentiation (LTP) is present in CA1 (black circle; *n* = 6) under control conditions and is disrupted under stressful conditions (open circles; *n* = 5).

We examined synaptic plasticity in more anterior locations of CA1 using a previously reported single-channel recording approach in an attempt to replicate previously reported disruptions in PPF and LTP (Fig. 4; Cazakoff and Howland 2010). Robust PPF was evident in CA1 (25–50 msec: 55.82 ± 8.78%; 100–200 msec: 18.29 ± 8.35%) for control animals (*n* = 6) but was disrupted in rats exposed to 30 min of restraint stress (25–50 msec: 24.74 ± 12.02; 100–200 msec: −4.06 ± 7.94%; *n* = 5; Fig. 4E). These impressions were confirmed by a significant main effect of Stress (*F*_1,9_ = 5.86, *P* = 0.04) and Interval (*F*_1,9_ = 45.06, *P* < 0.001) while significance was not achieved for Time of PPF or any interaction terms (statistics not shown). High-frequency stimulation of CA3 (100 Hz, 1 sec) also induced significant LTP in CA1 for controls (40.51 ± 7.31%), which was significantly disrupted following exposure to acute stress (18.40 ± 8.19%; Fig. 4F; *t*_(9)_ = 2.15, *P* = 0.03, one-tailed). Thus, the present results indicate that patterns of both short- and long-term plasticity are disrupted in the anterior CA1 region following exposure to acute stress, as previously demonstrated (Cazakoff and Howland 2010).

## Discussion

The present experiments report on the patterns of short- and long-term synaptic plasticity evoked simultaneously within CA1 and SUB using a novel in vivo dual-channel preparation in anesthetized rats (Fig. 1). Both CA1 and SUB fEPSPs displayed similar input/output profiles (Figs. 1D and 2B) to those previously reported (Cazakoff and Howland 2010; MacDougall and Howland 2013). In control rats, we demonstrate that stimulation of the CA3 region evokes fEPSPs in the CA1 and SUB characterized by significant PPF to stimulation intervals between 25 and 200 msec (Fig. 2). Application of a high-frequency tetanus induced strong LTP in both areas (Fig. 3). In contrast, LFS (1 Hz) did not affect evoked potentials in the CA1 while a late-developing potentiation was observed in SUB (Fig. 3). Exposure to acute stress immediately before anesthesia impaired LTP in the CA1 region and impaired PPF, LTP, and late-developing potentiation in SUB (Figs. 2, 3). A separate experiment using more anterior recording sites in CA1 confirmed the disruptive effect of acute stress on both PPF and LTP (Fig. 4). Taken together, our results highlight the potent effects of acute stress on synaptic plasticity within CA1 and SUB, the two major output structures of the hippocampal formation.

### Effects of acute stress on PPF in dorsal CA1 and SUB

In the dual-channel experiments, acute stress did not affect PPF in the CA1 while it was disrupted in the SUB before tetanic stimulation (Fig. 2). Previous research regarding the effects of acute stress on PPF in the CA1 region has been inconsistent. Research from our laboratory using CA1 recordings in vivo demonstrated a glucocorticoid receptor-dependent disruption in PPF following acute stress (Cazakoff and Howland 2010) while a previous study using hippocampal slices failed to observe altered PPF following acute stress (Shors and Thompson 1992). It is unknown why PPF was not disrupted in the present dual-channel experiments although it is worth noting that the CA1 recording site is 1.5 mm further posterior in the dual-channel preparation than the site used in our previous study (Cazakoff and Howland 2010). In addition, we confirm here that CA1 PPF is disrupted by acute stress in the more anterior recording site used previously (Fig. 4; Cazakoff and Howland 2010). Others have reported that the effects of acute stress on hippocampal LTP vary dramatically along the septotemporal axis with acute stress disrupting and enhancing LTP in the dorsal (septo) and ventral (temporal) CA1 regions, respectively (Maggio and Segal 2007; Segal et al. 2010). While it is possible that similar regional differences exist for the effects of acute stress on PPF, such an explanation is complicated by the disruption of CA1 LTP produced at the posterior CA1 recording site in our dual-channel experiments (Fig. 3). A rapidly induced (i.e., within minutes) reduction in CA1 PPF following administration of corticosterone to hippocampal slices has also been reported (Karst et al. 2005). This reduction in PPF depended on mineralocorticoid receptor activation, and may be the result of a nongenomic effect given its rapid occurrence (Karst et al. 2005).

Recordings from SUB revealed strong PPF in response to CA3 stimulation before the tetanus was delivered (Fig. 2). These findings compliment previous studies demonstrating PPF in the dorsal CA1-SUB pathway (Commins et al. 1998a, 2001; MacDougall and Howland 2013). Acute stress impaired SUB PPF similarly to previous experiments with the dorsal CA1-SUB pathway (Commins et al. 2001; MacDougall and Howland 2013). The present experiments did not confirm the previously reported reduction in PPF following the induction of LTP in the CA1-SUB pathway (Commins et al. 1998a; MacDougall and Howland 2013).

### Exposure to acute stress disrupts long-term potentiation in dorsal CA1 and SUB concurrently

Long-term potentiation is readily observed in both dorsal CA1 and SUB following stimulation of their primary monosynaptic inputs (i.e., CA3 and CA1, respectively; Behr et al. 2009; Malenka and Bear 2004). Our data demonstrate that LTP is reliably induced in both CA1 and SUB following HFS of CA3 (Fig. 3). Exposure to acute stress disrupted LTP in both CA1 and SUB concurrently (Fig. 3C), effects that may depend upon glucocorticoid receptor activation (Xu et al. 1998; Cazakoff and Howland 2010; MacDougall and Howland 2013). This result is consistent with numerous previous reports that acute stress impairs LTP in the CA3-CA1 pathway using different HFS protocols (Shors and Thompson 1992; Diamond and Rose 1994; Kim et al. 1996, 2005; Xu et al. 1998; Cazakoff and Howland 2010) and CA1-SUB pathway (Commins et al. 2001; MacDougall and Howland 2013). To our knowledge, we are the first to demonstrate LTP in SUB by stimulating CA3 and the first to induce LTP simultaneously from two synaptic loci within the hippocampal formation. Recordings of fEPSPs in freely moving rats have been conducted simultaneously in the dentate gyrus and basal amygdala in response to stimulation of entorhinal cortex (Yaniv et al. 2003; Vouimba et al. 2004). Using this preparation, acute stress was demonstrated to enhance early LTP in the basal amygdala without affecting early LTP in the dentate gyrus (Vouimba et al. 2004). Our data provide evidence that acute stress-induced disruptions in LTP occur in both CA1 and SUB, which opens the possibility that stress-induced disruptions in hippocampal-dependent spatial memory may be the result of concurrent disruptions in LTP within hippocampal formation output synapses rather than disruptions in CA1 LTP alone.

### Exposure to acute stress disrupts late-developing potentiation in SUB but has no effect on CA1 synaptic plasticity

Low-frequency stimulation of CA3 did not significantly alter fEPSPs recorded in the CA1 region; in contrast, late-developing potentiation was evident in SUB (Fig. 3). These results are consistent with previous reports suggesting that LFS does not induce synaptic plasticity in the CA1 of normal adult rats (Xu et al. 1997; Fox et al. 2007; Wong et al. 2007) while late-developing potentiation in SUB following LFS has been reported previously (Anderson et al. 2000; Huang and Kandel 2005; Fidzinski et al. 2008; MacDougall and Howland 2013). Exposure to acute stress did not induce the expected LTD in CA1 but disrupted the late-developing potentiation in SUB (Fig. 3). While LTD is typically enabled by acute stress in CA1 following LFS of CA3 (Xu et al. 1997; Fox et al. 2007; Wong et al. 2007), these previous studies have used a 3 Hz tetanus as compared to the 1 Hz tetanus used here. A 1 Hz tetanus was used as it induces a late-developing potentiation in SUB that was impaired by exposure to acute stress in a glucocorticoid receptor-dependent manner (MacDougall and Howland 2013). Here, we extend these findings to include LFS of the CA3 Schaffer collaterals. The divergent effects of LFS on CA1 and SUB plasticity suggest a unique role for SUB late-developing potentiation in learning and memory that is distinct from CA1 plasticity (Habib and Dringenberg 2010).

### Functional implications

Spatial cognition is mediated in part by mechanisms consistent with synaptic plasticity within the hippocampal formation (Martin et al. 2000; Malenka and Bear 2004; Whitlock et al. 2006; Wong et al. 2007; Collingridge et al. 2010; Klug et al. 2012). Exposure to acute stress exerts a profound impact on various forms of synaptic plasticity within the hippocampal formation both in vivo and in vitro and alters spatial learning and memory performance of a variety of hippocampal-dependent tasks (Kim and Diamond 2002; Joels et al. 2006; Diamond et al. 2007; Howland and Wang 2008; Cazakoff et al. 2010; Collingridge et al. 2010). Importantly, both the dorsal CA1 and subiculum are involved in processing spatial information and memory (Morris et al. 1990; McNaughton et al. 1996; O'Mara et al. 2009); therefore, it is reasonable to conclude that the impairments in synaptic plasticity in both output regions of the hippocampal circuit contribute to the deficits in spatial memory retrieval observed following acute stress (Cazakoff et al. 2010; MacDougall and Howland 2013; O'Mara et al. 2009). However, anatomical and behavioral data suggest that their roles in spatial cognition are likely distinct (Behr et al. 2009). While the dorsal CA1 receives strong input from the CA3 subregion through glutamatergic Schaffer collaterals and other input from the cortex via the temporoammonic pathway (Behr et al. 2009), the subiculum receives prominent projections from the CA1 (Amaral et al. 1991) and cortical areas including the entorhinal, perirhinal, and postrhinal areas (Naber et al. 2001b; Behr et al. 2009; O'Mara et al. 2009). Thus, the subiculum is in a privileged position to receive both highly processed information from the hippocampus and “raw” sensory information directly from the cortex (Behr et al. 2009). Behavioral experiments have shown delay-dependent changes in contribution of the neural activity in the CA1 and subiculum to encoding information during a spatial delayed-nonmatch-to-sample task (Deadwyler and Hampson 2004). Therefore, determining whether the specific demands of a given task dissociate the involvement of the acute stress effects on CA1 and subicular synaptic plasticity in spatial cognition will be the subject of future investigation.
